# Global Prevalence of *Entamoeba histolytica* in Children: A Systematic Review and Meta‐Analysis

**DOI:** 10.1002/hsr2.73453

**Published:** 2026-10-27

**Authors:** Nader Salari, Avijeh Rahimi, Seyed Hamidreza Hashemian, Mojtaba Ammari‐Allahyari, Shamarina Shohaimi, Masoud Mohammadi

**Affiliations:** ^1^ Department of Biostatistics School of Health, Kermanshah University of Medical Sciences Kermanshah Iran; ^2^ Student Research Committee Kermanshah University of Medical Sciences Kermanshah Iran; ^3^ Faculty of Mathematics and Data Science Emirates Aviation University Dubai UAE; ^4^ Department of Biology Faculty of Science, University Putra Malaysia Serdang Malaysia; ^5^ Research Center for Social Determinants of Health Jahrom University of Medical Sciences Jahrom Iran

**Keywords:** amebiasis, children, *Entamoeba histolytica*, meta‐analysis

## Abstract

**Background:**

Infection with *Entamoeba histolytica* in children can seriously affect individual and public health. Moreover, the prevalence of this disease varies significantly across different regions of the world, and comprehensive and accurate data on its global prevalence among children are lacking. The present study aims to determine the global prevalence of *Entamoeba histolytica* in children.

**Methods:**

This study is a systematic review and meta‐analysis of existing evidence on the global prevalence of *Entamoeba histolytica* in children, conducted according to the PRISMA 2020 statement criteria. The search was conducted using the (AND) and (OR) operators, and the retrieved data were transferred to the EndNote reference management software. The quality assessment of the studies included in the study was conducted based on the STROBE checklist, considering the nature of the cross‐sectional studies included in the study. I^2^ index was used to evaluate heterogeneity. Funnel Plot and Begg and mazumdar correlation test were used to evaluate the publication bias. Data were analyzed using Comprehensive Meta‐Analysis software (Version 2).

**Results:**

A total of 119 studies with a sample size of 70,259 children were included. The *I* ^2^ heterogeneity test indicated high heterogeneity (*I* ^2^: 97.7%); therefore, a random‐effects model was used for data analysis. Based on the meta‐analysis, the global prevalence of *Entamoeba histolytica* in children was reported as 8.4% (95% CI: 7.1–10). However, given the results of the publication bias test, the results should be considered with caution, and it is also stated that the geographically heterogeneous distribution of the studies is also a limitation of this study. the pooled prevalence estimate should be interpreted with caution despite the subgroup and meta‐regression analyses performed.

**Conclusion:**

According to the results of the meta‐analysis, the global prevalence of Entamoeba histolytica in children was reported as 8.4%; therefore, although the final prevalence is relatively high, it should be investigated and considered in light of the transmissibility of this disease in children and its impact.

## Highlights

- The search was conducted using the (AND) and (OR) operators without time restrictions on December 30, 2024.
- A total of 119 studies with a sample size of 70,259 children were included.
- The I^2^ heterogeneity test indicated high heterogeneity (*I* ^2^: 97.7%).
- The global prevalence of *Entamoeba histolytica* in children was reported as 8.4%.

**Abbreviations**
PRISMA: The Preferred Reporting Items for Systematic Reviews and Meta‐Analyses
STROBE: The Strengthening the Reporting of Observational Studies in Epidemiology

## 1 Background

Amebiasis is defined as a gastrointestinal infection caused by *E. histolytica* with or without clinical manifestations [1], the genus Entamoeba includes at least seven different species including *E. histolytica*, *E. coli*, *E. hartmanni, E. polecki*, *E. dispar*, *E. moshkovskii* and E. *bangladeshi*, the species known to cause invasive amebiasis is Entamoeba histolytica, which affects the large intestine [1, 2, 3].

Individuals of all ages can be affected by *E. histolytica*; however, children are at a higher risk due to their immature immune systems and poor personal hygiene practices [2, 3, 4, 5]. Transmission can occur directly through the fecal‐oral route via person‐to‐person contact or indirectly through consuming contaminated food, raw vegetables, or water containing human feces. The cysts of this parasite are highly resistant and can survive in moist soil for weeks [6, 7].

Symptoms of the disease include fever, chills, vomiting, loss of appetite, abdominal pain, and bloody or mucus‐filled diarrhea alternating with periods of constipation [8, 9]. Bloody diarrhea occurs when E. histolytica trophozoites invade and ulcerate the large intestinal wall. In extra‐intestinal cases, the parasite can spread to various organs, including the lungs, brain, and liver, leading to abscess formation. If left untreated, this condition can result in serious and even fatal complications [10].

Although the mortality rate associated with *Entamoeba histolytica* infection is relatively low compared to other infectious diseases, the long‐term impact of this infection can pose serious public health challenges [2].

Children are particularly vulnerable to the harmful effects of this infection, as they are in a critical phase of high metabolism, rapid physical growth, and learning development [8, 11]. Diagnosing protozoan infections like *E. histolytica* requires collecting at least three stool samples on three different days to ensure the accuracy of results [12, 13, 14]. To improve school health and reduce the spread of infections, UNICEF has outlined three key strategies: Handwashing with soap, providing accessible drinking water and Establish separate toilets for boys and girls and ensure daily sanitation [15, 16]. According to studies, *E. histolytica* infection is widespread in developing countries such as Ethiopia and Nepal, as well as in tropical regions like the Sahara in Africa and parts of Asia. This situation highlights the urgent need for greater attention to public health issues and preventive measures in these areas [1, 4, 6, 17]. Given the higher prevalence of *E. histolytica* infection in children and its significant consequences in this age group, this systematic review and meta‐analysis aim to provide an overview of amebiasis in children and offer a general estimate of the global prevalence of the *E. histolytica* parasite in children.

## 2 Methods

This study is a systematic review and meta‐analysis of existing evidence on the global prevalence of *Entamoeba histolytica* in children, conducted following the PRISMA 2020 statement guidelines. The review period is from January 1981 to December 30, 2024. Given that the study was a review and the age range for children is important, we have reported it in Table 1, but for us, the search title included the word “children” in the study itself, and any study that had the title “children” was included in the review.

**Table 1:** Summary of the prevalence studies included in the review.

author	year	region	Sample size	Range age	Prevalence *N* (%)	Quality assessment
Muhajir et al. [18]	2017	Sudan	200	6–18	31 (15.5)	Moderate
Abed et al. [19]	2015	Iraq	200	< 10	55 (27.5)	Moderate
Aher et al. [20]	2011	India	624	6–12	24 (3.9)	Moderate
Ahmed et al. [21]	2010	Sudan	157	6–18	31 (19.7)	Moderate
Ahmed et al. [13]	2023	Iraq	247	2–13	94 (38)	High
Wadood et al. [22]	2005	Pakistan	220	< 13	64 (29)	Moderate
Ajayi et al. [23]	2017	Nigeria	386	3–14	138 (35.8)	Moderate
Aksoy et al. [24]	2007	Turkey	1127	7–14	2 (0.18)	Moderate
Al Saadawy et al. [25]	2018	Egypt	600	6–12	7 (1.17)	High
Afrah et al. [14]	2020	Iraq	680	1–13	198 (29.11)	High
Al‐Ballaa et al. [26]	1993	Saudi Arabia	8000	< 6	218 (2.7)	Moderate
Al‐Braiken [27]	2008	Saudi Arabia	500	< 10	30 (6)	Moderate
Al‐Dammy et al. [15]	2020	Iraq	100	< 10	9 (9)	High
Al‐Mekhlafi et al. [28]	2016	Yemen	1218	5–15	262 (21.5)	High
Al‐Zain [29]	2009	Palestine	256	2–15	13 (5.1)	Moderate
Tabeena Ali et al. [8]	2024	Pakistan	160	< 2	28 (17.5)	Moderate
Alsubaie et al. [30]	2015	Yemen	258	8–15	87 (33.7)	Moderate
Apaza et al. [2]	2023	Bolivia	790	5–13	17 (2.2)	Moderate
Asai et al. [31]	2016	Bolivia	2033	2–18	191 (9.39)	Moderate
Aschale et al. [16]	2021	Ethiopia	407	10–12	19 (4.67)	Moderate
Ashok et al. [32]	2013	India	208	—	41 (19.71)	Moderate
Assavapongpaiboon et al. [33]	2018	Thailand	263	4–15	3 (1.1)	Moderate
Assudani et al. [34]	2015	India	180	1–10	6 (3.33)	Moderate
Astal [35]	2004	Palestine	1370	6–11	96 (7)	Moderate
Banhos et al. [36]	2017	Brazil	367	4–12	51 (13.9)	Moderate
Barwari et al. [37]	2011	Iraq	800	< 1–14	95 (11.9)	Moderate
Basima et al. [38]	2005	Iraq	350	< 5–14	22 (6.3)	Moderate
Bauhofer et al. [9]	2020	Mozambique	1004	< 14	20 (2)	Moderate
Bayoumy et al. [39]	2016	Egypt	1615	6–16	223 (13.8)	Moderate
Bhattachan et al. [40]	2015	Nepal	296	1–18	8 (2.7)	Moderate
Bhattachary et al. [41]	2017	India	1000	1–12	219 (21.9)	Moderate
Bisetegn et al. [6]	2023	Ethiopia	450	6–17	42 (9.3)	Moderate
Bisht et al. [42]	2011	India	335	< 14	26 (7.76)	Moderate
Canete et al. [43]	2012	Cuba	104	< 5	2 (1.9)	Moderate
Chandi et al. [44]	2018	India	250	6–15	30 (12)	High
Dagne et al. [7]	2021	Ethiopia	280	5–15	70 (25)	High
Dana et al. [45]	2016	Ethiopia	371	—	16 (4.31)	Moderate
Debash et al. [4]	2023	Ethiopia	402	4–16	52 (12.93)	High
Deka et al. [17]	2022	India	123	< 5	9 (7.3)	High
Edosomwan et al. [46]	2020	Nigeria	184	6–15	125 (67.93)	High
Eisa et al. [47]	2019	Sudan	100	< 12	2 (2)	High
Elmonir et al. [3]	2021	Egypt	996	1–15	126 (12.7)	High
Erismann et al. [48]	2016	Burkina Faso	385	8–14	256 (66.5)	Moderate
Evbuomwan et al. [49]	2022	Nigeria	249	6–15	160 (64.3)	Moderate
Faraji et al. [50]	2015	Iran	632	0 – 6	22 (3.48)	Moderate
Faulkner et al. [51]	2003	Mexico	438	< 16	1 (0.22)	Moderate
Feleke et al. [52]	2022	Ethiopia	375	< 7– < 14	4 (1)	Moderate
Gadisa et al. [53]	2019	Ethiopia	561	< 5	33 (5.9)	Moderate
Gebretsadik et al. [54]	2020	Ethiopia	400	7−14	33 (8.3)	Moderate
Gelaw et al. [55]	2013	Ethiopia	304	< 9–13 <	28 (9.21)	Moderate
Getnet et al. [12]	2022	Ethiopia	384	5–16	19 (4.9)	High
Gupta et al. [56]	2020	Nepal	285	5–15	22 (7.7)	High
Gyang et al. [57]	2019	Nigeria	384	7–17	97 (25.3)	Moderate
Hafeez et al. [58]	2018	Pakistan	708	1–14	23 (3.24)	Moderate
Hailegebriel [59]	2017	Ethiopia	359	7–14	88 (24.5)	Moderate
Hailu et al. [11]	2021	Ethiopia	465	6–15	117 (18.1)	Moderate
Heidari et al. [60]	2003	Iran	461	< 6	11 (2.4)	Moderate
Ayman [61]	2011	Palestine	735	—	71 (9.7)	Moderate
Amjed et al. [62]	2012	Iraq	1520	< 12	149 (9.8)	Moderate
Abdulkhaleq et al. [63]	2011	Iraq	730	< 18	103 (14)	Moderate
Ibrahim et al. [64]	2018	Libya	156	< 14	20 (12.8)	Moderate
Ibrahim et al. [1]	2020	nigeria	120	7–15	60 (50)	High
Ihejirika et al. [65]	2019	nigeria	300	5–13	16 (5.3)	High
AL‐Rubaee [66]	2008	Iraq	252	6–11	14 (5.5)	Moderate
Jarallah et al. [67]	2012	Iraq	294	1–14	80 (27.2)	Moderate
Kadir et al. [68]	2018	Iraq	1100	6–12	102 (9.3)	Moderate
Khan et al. [69]	2022	Pakistan	184	10–17	10 (5.43)	
Kiani et al. [70]	2016	Iran	500	—	1 (0.2)	Moderate
Kurup et al. [71]	2010	Saint Lucia	554	< 19	7 (1.26)	Moderate
Legesse et al. [72]	2010	Ethiopia	386	7–18	7 (1.8)	Moderate
Liao et al. [73]	2017	Taiwan	308	6–16	54 (17.53)	Moderate
Malatyalı et al. [74]	2011	Turkey	1449	6–15	22 (1.51)	Moderate
Matthys et al. [75]	2011	Tajikistan	594	7–11	154 (25.9)	Moderate
Mbae et al. [76]	2013	Kenya	2112	< 5	225 (10.7)	Moderate
Mekonnen et al. [77]	2019	Ethiopia	310	< 5	21 (6.8)	High
Mohammed et al. [78]	2022	Ethiopia	221	< 5	24 (10.86)	High
Mukhiya et al. [79]	2012	Nepal	342	—	7 (2)	Moderate
Mukhtar et al. [80]	1981	Nepal	182	4–10	10 (5.5)	Moderate
Mulatu et al. [81]	2015	Ethiopia	158	< 5	18 (11)	Moderate
Mutungi et al. [82]	2019	Kenya	481	7–14	113 (23.5)	High
Ndifor et al. [83]	2020	Cameroon	200	< 7	63 (31.5)	High
Ndifor et al. [84]	2021	Cameroon	200	< 5	11 (5.5)	High
Neamah et al. [85]	2023	Iraq	427	< 10	91 (21.31)	High
Nguyen et al. [86]	2012	Ethiopia	664	6−19	177 (26.7)	Moderate
Nitiema et al. [87]	2011	Burkina Faso	309	< 5	4 (1.3)	Moderate
Njambi et al. [88]	2020	Kenya	180	8–14	22 (12.2)	Moderate
Njunda et al. [89]	2015	Cameroon	411	—	9 (2.18)	Moderate
Obadiah [90]	2011	Nigeria	183	< 5	78 (42.62)	Moderate
Obala et al. [91]	2013	Kenya	797	< 5	86 (10.79)	Moderate
Padmaja et al. [92]	2014	India	200	—	62 (31)	Moderate
Patel et al. [93]	2006	Oman	436	9−10	105 (24.1)	Moderate
Qadri et al. [94]	1995	Saudi Arabia	3258	6−11	24 (0.74)	Moderate
Rai et al. [95]	2017	Nepal	359	4–16	24 (6.68)	
Reji et al. [96]	2011‘	Ethiopia	358	6–17	45 (12.6)	Moderate
Sah et al. [97]	2013	Nepal	200	—	16 (8)	Moderate
Sakari et al. [98]	2017	Kenya	361	≥ 2 to ≤ 5	1 (0.28)	Moderate
Sejdini et al. [99]	2011	Albania	321	< 16	1 (0.3)	Moderate
Sharma et al. [100]	2004	Nepal	533	4–19	38 (7.1)	Moderate
Shiferaw et al. [101]	2021	Ethiopia	384	7–18	18 (4.7)	High
Shrestha et al. [10]	2021	Nepal	400	—	3 (0.75)	High
Shrestha et al. [102]	2018	Nepal	770	< 5	17 (2.2)	Moderate
Shubha et al. [103]	2011	India	1224	6–12	94 (7.7)	Moderate
Singh et al. [104]	2015	India	100	—	5 (5)	Moderate
Suliman et al. [105]	2019	Sudan	253	10−17	79 (31.2)	Moderate
Tadesse et al. [106]	2019	Ethiopia	417	6–16	43 (10.3)	Moderate
Taher et al. [107]	2023	Iraq	218	< 12	39 (17.9)	High
Tandukar et al. [108]	2013	Nepal	1392	5–15	47 (3.38)	Moderate
Tenali et al. [109]	2018	India	1246	5–18	121 (9.7)	Moderate
Thapa et al. [110]	2011	Nepal	279	4–15	30 (10.75)	Moderate
Tulu et al. [111]	2014	Ethiopia	340	6–14	17 (5)	Moderate
Verma et al. [112]	2017	India	351	3–12	25 (7.1)	Moderate
Vilwanathan et al. [113]	2017	India	204	≤ 12	17 (8.33)	Moderate
Wadile et al. [114]	2013	India	100	5–10	20 (20)	Moderate
Waikagul et al. [115]	2002	Thailand	1010	—	14 (1.4)	Moderate
Warunee et al. [116]	2007	Thailand	1920	7–12	5 (0.3)	Moderate
Wasihun et al. [5]	2020	Ethiopia	610	< 5	125 (20.5)	High
Yeshitila et al. [117]	2020	Ethiopia	312	≤ 9–15 <	34 (10.9)	High
Yin et al. [118]	2022	Eswatini	316	5−15	10 (3.2)	High
Zemene et al. [119]	2018	Ethiopia	247	< 5	14 (5.7)	Moderate

The search was conducted using the keywords: “prevalence,” “*Entamoeba histolytica*,” “Acanthamoeba infection,” “amebiasis, “parasitic infection,” and “children”, utilizing the (OR) and (AND) operators.

To identify relevant studies, searches were performed in six significant databases: Web of Science, Google Scholar, Scopus, ScienceDirect, PubMed, Embase.

PubMed search sample: ((((((prevalence[Title/Abstract]) AND (Entamoeba histolytica[Title/Abstract])) OR (Endamoeba histolytica[Title/Abstract])) OR (Acanthamoeba infection[Title/Abstract])) OR (Amebiasis[Title/Abstract])) OR (Amebiasis[Title/Abstract])) OR (parasitic infection[Title/Abstract])) AND (children[Title/Abstract])) OR (child[Title/Abstract]))))))

Additionally, reference lists and citations in each retrieved article were examined to locate related studies. No restrictions were applied regarding the publication year. The last update to the searches was conducted on December 30, 2024, and only studies that reported the global prevalence of *Entamoeba histolytica* in children according to the inclusion criteria were considered.

### 2.1 Inclusion and Exclusion Criteria

Inclusion criteria: studies reporting the prevalence of *Entamoeba histolytica* in children; studies that explicitly specify the pediatric age group (using the term “children”); studies for which the full text is available; studies that provide sufficient data (sample size and prevalence percentage); studies published in English; and cross‐sectional studies.

Exclusion Criteria: Review studies (descriptive observational studies). Interventional studies. Cohort studies. Case‐control studies. Duplicate studies. Studies not published in English. Studies with insufficient data (lack of sample size or prevalence percentage). Studies where full‐text was unavailable.

### 2.2 Study Selection

The EndNote software was used to organize and manage the studies systematically. The selection process followed the PRISMA 2020 guidelines for systematic reviews.

780 articles were identified through database searches, along with one additional potentially relevant article found through manual searching. These articles were imported into the EndNote reference management software. After removing 185 duplicate articles, the remaining studies underwent a three‐stage evaluation conducted by two independent researchers. In cases of disagreement, a third reviewer resolved the differences. Screening Stage: The titles and abstracts of all retrieved studies were assessed based on the inclusion and exclusion criteria, which resulted in the removal of 312 studies. Eligibility Assessment: The full‐text articles of the remaining studies were reviewed, and 164 studies were excluded due to a lack of accessible data or low quality. Final Selection: A total of 120 studies were included in this systematic review.

### 2.3 Quality Assessment

A checklist for observational studies was used to validate and assess the methodological quality of the included studies. The Strengthening the Reporting of Observational Studies in Epidemiology (STROBE) checklist, which consists of six key sections: Title, Abstract, Introduction, Methods, Results, and Discussion, was applied.

The STROBE checklist consists of 32 items, covering essential methodological aspects such as: Study title and problem statement, Study objectives and type, Study population and sampling methods, Sample size determination, Definition of variables and procedures, Data collection tools, Statistical analysis methods, Findings and discussion. Studies were classified based on methodological quality: Studies scoring 16 or higher were considered suitable to moderate methodological quality and included in the review. Studies scoring below 16 were considered methodologically weak and were excluded from the analysis.

### 2.4 Data Extraction

Data extraction was performed by two independent researchers using a pre‐designed checklist. This checklist included one main page for extracting global prevalence data and five additional pages for analyzing prevalence across different continents, including Africa, Asia, South America, North America, and Europe. The extracted data encompassed various aspects such as the first author's name, year of publication, type of study, study location, combined total sample size for both genders, and separate sample sizes for boys and girls. It also included the age range, the number and prevalence percentage for both genders combined, the number and prevalence percentage for boys and girls separately, and the type of laboratory sample used for diagnosis.

### 2.5 Statistical Analysis

Data were analyzed using Comprehensive Meta‐Analysis software (Version 2). Funnel Plot and Begg and mazumdar correlation test were used to evaluate the publication bias. Meta‐regression test was used to investigate the effective factors in creating heterogeneity in meta‐analysis results. I^2^ index was used to evaluate heterogeneity.

## 3 Results

In total, 780 studies were identified through searches across six databases, and one additional study was obtained by reviewing other sources (including the reference lists of the identified articles), resulting in a total of 781 items. After removing 186 duplicates, the titles and abstracts of the remaining articles were carefully screened, yielding 595 articles. Subsequently, based on the established criteria, 312 of these were excluded, leaving 283 articles to proceed to the next stage. In the following phase, the full texts of these articles were reviewed against the inclusion and exclusion criteria, leading to the exclusion of 164 articles. Ultimately, 119 articles were selected for the study and meta‐analysis (Figure 1 and Table 1).

**Figure 1:**
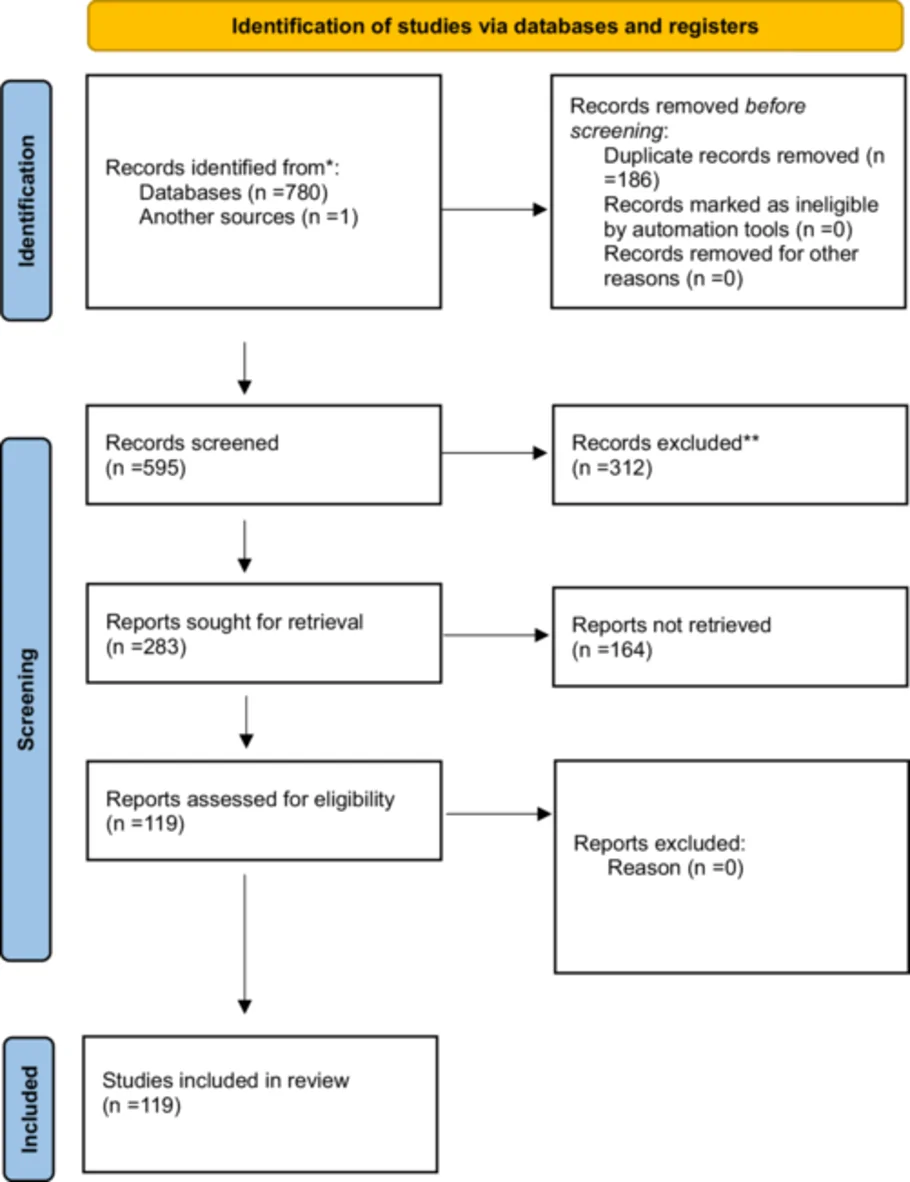
Flow chart (PRISMA 2020) indicating the stages of article selection in this systematic review and meta‐analysis.

An analysis of 119 studies, comprising a total sample of 70,259 children, revealed a high level of heterogeneity based on the *I* ^2^ test, with an *I* ^2^ value of 97.7%. Due to this substantial heterogeneity, a random‐effects model was employed to analyze the results. According to the meta‐analysis, the global prevalence of *Entamoeba histolytica* infection in children was estimated at 8.4%, (95% CI: 7.1–10, 0.000), as shown in Figure 2. Additionally, an assessment of publication bias using Begg and Mazumdar's correlation test indicated the presence of publication bias, with a p‐value of 0.000 (*p* < 0.001), (Figure 3). The pooled prevalence estimate should be interpreted with caution despite the subgroup and meta‐regression analyses performed. The results of the leave‐one‐out sensitivity analysis are reported in Figure 4, which does not show any significant changes resulting from the examination of each study (Figure 4).

**Figure 2:**
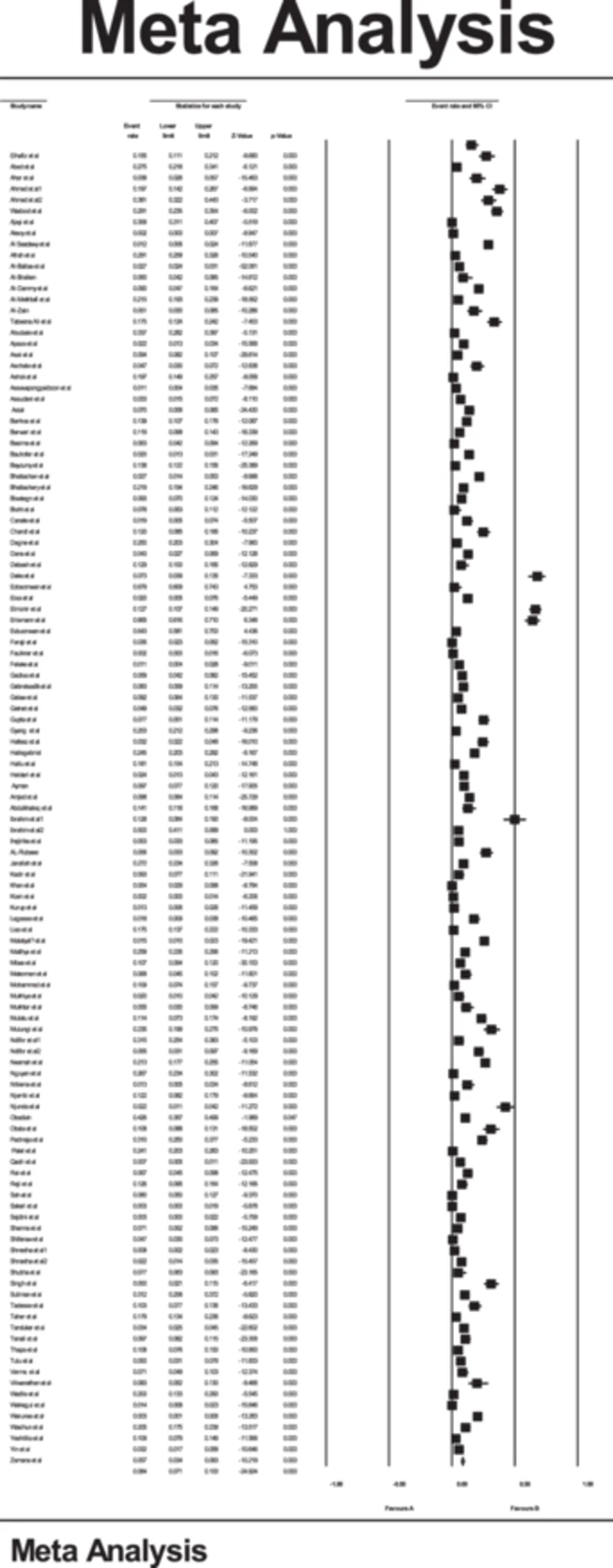
Forest plot of the global prevalence of *Entamoeba histolytica* in children based on the random effect's method.

**Figure 3:**
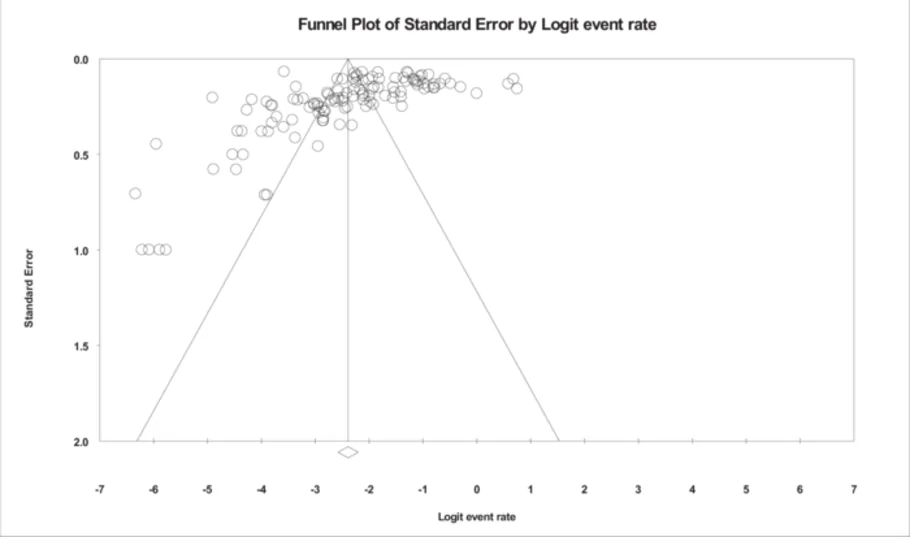
Funnel plot assessing publication bias in the reviewed studies.

**Figure 4:**
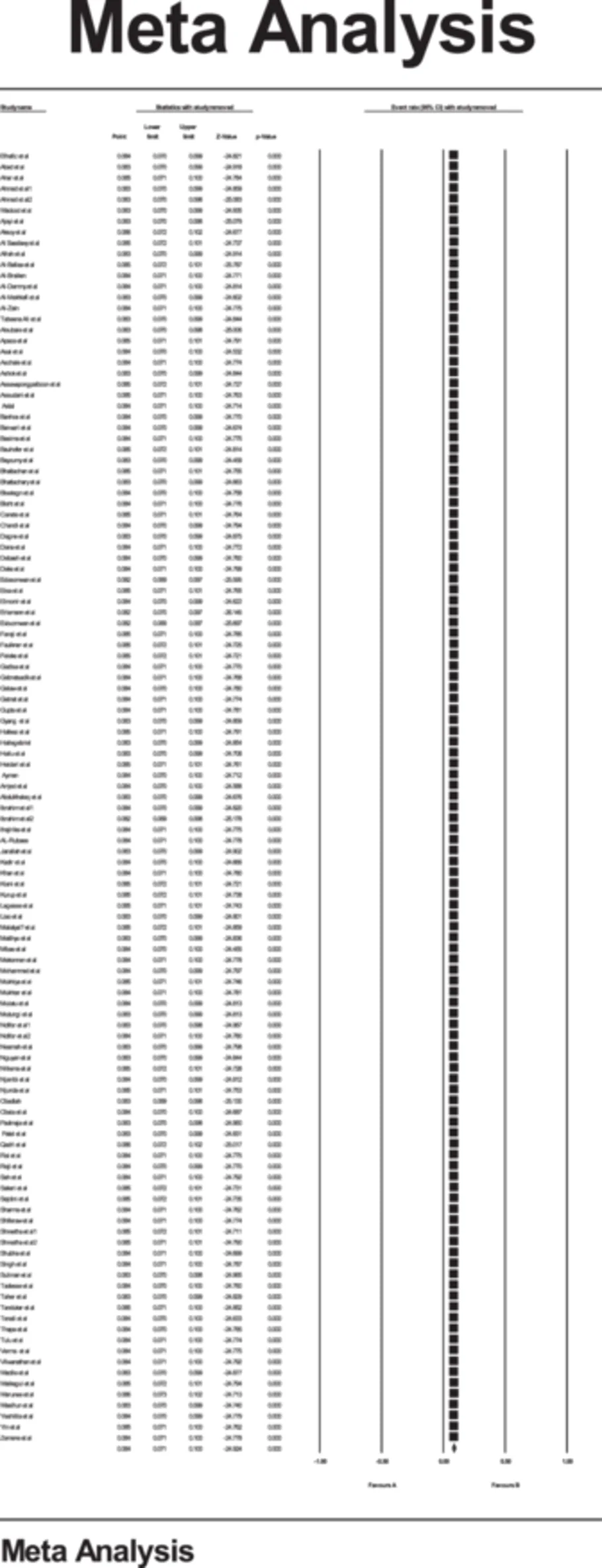
Report on the Results of Sensitivity Analysis in the Reviewed Studies.

In examining the factors influencing study heterogeneity and assessing the impact of sample size on this heterogeneity, it was reported that as the sample size increases, the global prevalence of *Entamoeba histolytica* in children decreases (*p* < 0.05) (Figure 5A). Additionally, as the year of study increases, the global prevalence of *Entamoeba histolytica* in children increases (*p* < 0.05) (Figure 5B).

**Figure 5:**
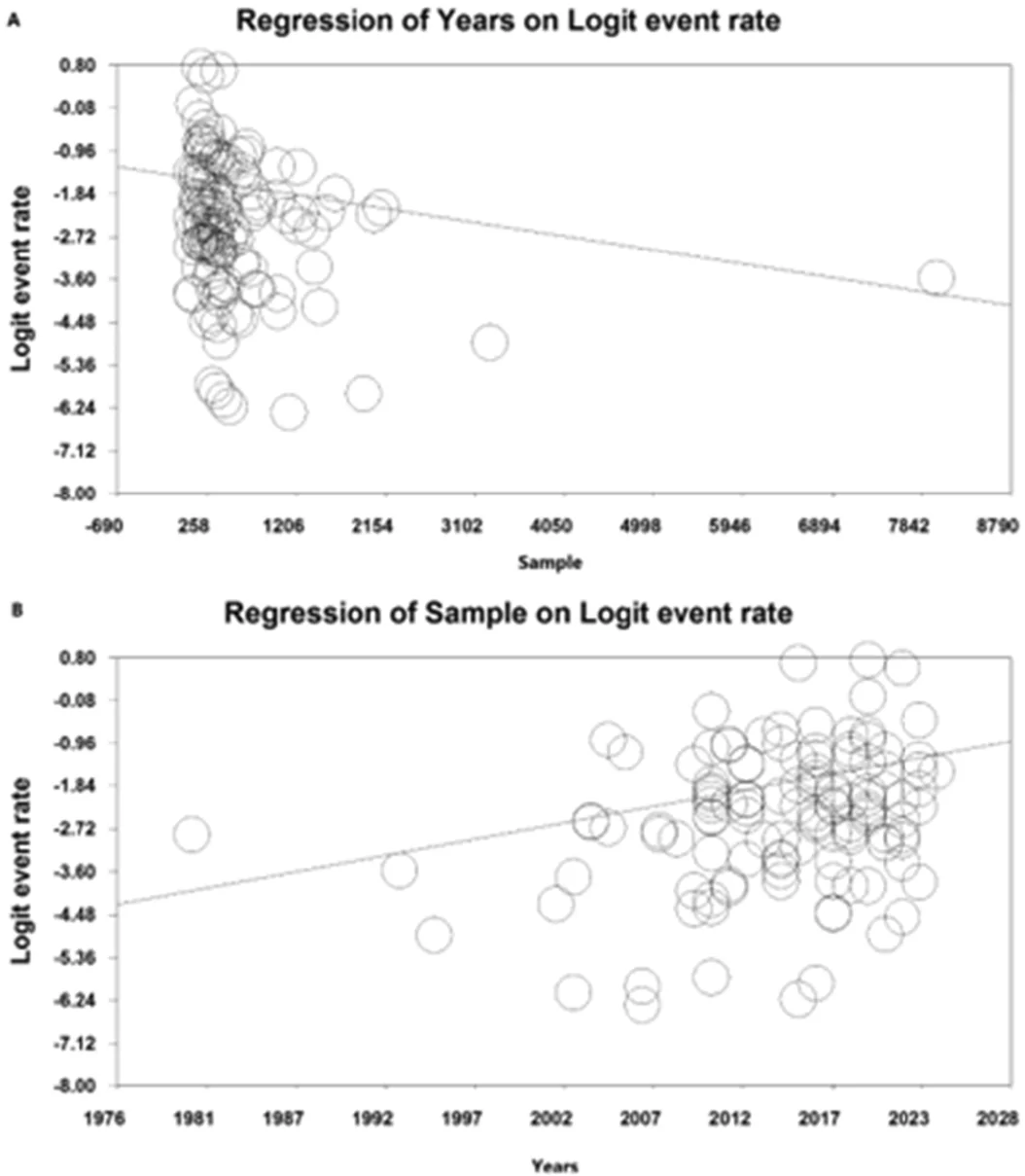
(A) Meta‐regression of the impact of sample size on the global prevalence of *Entamoeba histolytica* in children. (B) Meta‐regression of the impact of study year on the global prevalence of *Entamoeba histolytica* in children.

Based on the results of Table 2, which reports the global prevalence of *Entamoeba histolytica* in children by continent, the highest prevalence was reported in Africa at 11.2% (95% CI: 8.7–14.3), while the lowest prevalence was observed in North America at 1.1% (95% CI: 0.4–2.6). Since only one study was reported in Europe, no analysis was conducted based on that single study.

**Table 2:** Global Prevalence of *Entamoeba histolytica* in children by continent.

Continent	*N*	Sample size	*I* ^2^	Begg and mazumdar correlation test	Prevalence (95% CI)
Africa	51	21984	97.7	0.0001	11.2 (95% CI: 8.7–14.3)
Asia	61	43668	97.7	0.0001	7.4 (95% CI: 5.8–9.3)
North America	3	1096	38.1	1.0000	1.1 (95% CI: 0.4–2.6)
South America	3	3190	95.8	1.0000	6.9 (95% CI: 3.2–14.3)

## 4 Discussion

Infection with *Entamoeba histolytica* (EH) can lead to amebiasis, which, if not treated in a timely manner, poses a serious threat to children's health. This study, using a systematic review and meta‐analysis approach, examined the global prevalence of *E. histolytica* in children. Analyzing 119 studies with a total sample size of 70,259 children from 1981 to 2024, the overall prevalence of *E. histolytica* in children was found to be 8.4%, highlighting a significant public health concern worldwide.

One of the main causes of *E. histolytica* infection is poor hygiene practices. Key factors such as not trimming nails, failing to wash hands after playing and before eating, and neglecting personal hygiene all contribute to the increased prevalence of this parasite [4, 7, 8, 28, 55].

Our findings indicate that the highest prevalence of the *Entamoeba histolytica* (*E. histolytica*) parasite in children is in Africa. Analyzing 51 studies with a total sample size of 21,984 children, the prevalence of *E. histolytica* was found to be 11.2%. Several factors contribute to this situation in the continent, including contaminated water sources, low living standards, poor personal and environmental hygiene, and inadequate sanitation among food vendors [1, 46, 49, 57, 90].

In the analysis of 61 studies with a total sample size of 43,668 children in Asian countries, the prevalence of *Entamoeba histolytica* was found to be 7.4%. However, this prevalence varies across different Asian countries. These differences are influenced by factors such as geographical region, economic conditions, and social and cultural aspects [14, 24, 60]. In low‐income and tropical countries, the prevalence of the parasite is higher than in other regions. This could be attributed to various factors, including the lack of effective medications for treating infections, lower levels of awareness and education, inadequate sanitation and healthcare facilities, and limited access to purified drinking water in all schools [8, 14, 24, 33, 60, 69].

Several factors contribute to this lower prevalence, including greater access to safe drinking water and better hygiene standards, which are more prevalent in high‐income and developed countries compared to low‐income and developing nations. These differences may explain the lower incidence of parasitic infections in this region [31, 43].

Special attention to children's hygiene, proper parental education in schools, and the timely diagnosis and treatment of infected individuals have significantly contributed to reducing the prevalence of intestinal parasites in Latin American countries and some Asian nations. These measures could serve as effective models for improving public health conditions in other regions as well [43, 71, 120].

Maintaining hygiene among children is very important to prevent this parasitic disease. The activities of young children who spend more time outdoors and in playgrounds, are constantly in contact with contaminated soil, do not consistently observe hand hygiene, and often eat with unwashed hands, highlight the importance of hygiene among children to prevent this disease [1, 2, 46, 49, 55, 70].

Meta‐regression results indicate that the prevalence of the condition has increased over the years—contrary to the expectation that it would decline over time due to scientific and technological advancements. This trend may stem from the varying impact of such advancements across different societies, as well as the disease's prevalence in less developed regions; indeed, subgroup analyses reveal a higher concentration of studies from Africa and Asia compared to other parts of the world, suggesting that the disease's prevalence is influenced by levels of development and the limited reach of scientific progress in these communities.

The heterogeneity reported in this study indicates high heterogeneity in studies, which is why we used the random effects method to examine the meta‐analysis. This heterogeneity could be due to differences in gender, age, differences in samples, differences in geographical region and race, and differences in the year of study. In our study, based on what the studies reported, we used meta‐regression to examine this heterogeneity in two areas: sample size and year of study, which showed that the prevalence of *Entamoeba histolytica* decreases with increasing sample size and increases with increasing year of study.

The findings of this study clearly highlight that the prevalence of *E. histolytica* infection in children is a serious public health challenge requiring urgent attention and intervention. Given the high prevalence in certain countries, it is essential for educational institutions and policymakers to design and implement effective intervention programs. These programs could include enhanced health education in schools, increased awareness among families, ensuring a permanent supply of soap in school restrooms, improving the sanitation of available water sources, providing access to safe drinking water, and creating clean and safe play environments for children. Additionally, considering cultural factors and regional conditions can help develop more effective and context‐specific solutions.

Although this study analyzed 120 articles from 30 countries reporting the prevalence of *E. histolytica* in children, there remains a lack of data from broader geographical regions worldwide. Given these data gaps in certain areas, further research is necessary to obtain a more comprehensive global picture of *E. histolytica* prevalence and to implement effective measures for its prevention and control.

### 4.1 Strengths and Limitations

The key strengths of this study lie in the comprehensive search of databases to gather all relevant data for a global review, as well as the conduct of meta‐regression and subgroup analyses, which provide a precise and reliable estimate of prevalence. One limitation of this study is the high heterogeneity among the studies, which is why we used the random‐effects model for the meta‐analysis. However, when examining other information from the reviewed studies, appropriate information for subgroup analyses by geography, gender, and other factors was not obtained, and this information was not obtained consistently across all studies. The geographical distribution of the studies also indicates that the distribution of studies is not uniform around the world. Although we conducted subgroup analyses for continents, there is still a heterogeneous distribution of studies across continents. Another limitation of this study is the significance of the Bagg test in terms of publication bias, which recommends using the data with caution and reporting the bias meaningfully.

## 5 Conclusion

The findings of this study indicate a relatively high prevalence of Entamoeba histolytica in studies conducted worldwide. Therefore, given this prevalence, it is necessary to consider this disease in children with greater attention. These findings can play a crucial role in shaping health policy, guiding care practices, and informing public education across health centers, social media platforms, and schools thereby enabling more precise preventive measures against this disease for families and children.

## Author Contributions

**Nader Salari:** conceptualization, writing – original draft. **Avijeh Rahimi:** writing – original draft, investigation. **Seyed Hamidreza Hashemian:** investigation, writing – original draft. **Mojtaba Ammari‐Allahyari:** writing – review and editing, supervision. **Shamarina Shohaimi:** writing – review and editing, supervision. **Masoud Mohammadi:** conceptualization, investigation, methodology, software, supervision, project administration, writing – review and editing, writing – original draft.

## Ethics Statement

The authors have nothing to report.

## Conflicts of Interest

The authors declare no conflicts of interest.

## Transparency Statement

The Corresponding author Masoud Mohammadi affirms that this manuscript is an honest, accurate, and transparent account of the study being reported; that no important aspects of the study have been omitted; and that any discrepancies from the study as planned (and, if relevant, registered) have been explained.

## Data Availability

The data that support the findings of this study are available from the corresponding author upon reasonable request. Datasets are available through the corresponding author upon reasonable request.
